# *Chlamydia trachomatis* inclusion membrane protein MrcA interacts with the inositol 1,4,5-trisphosphate receptor type 3 (ITPR3) to regulate extrusion formation

**DOI:** 10.1371/journal.ppat.1006911

**Published:** 2018-03-15

**Authors:** Phu Hai Nguyen, Erika I. Lutter, Ted Hackstadt

**Affiliations:** 1 Host-Parasite Interactions Section, Laboratory of Bacteriology, National Institute of Allergy and Infectious Diseases, National Institutes of Health, Hamilton, Montana, United States of America; 2 Department of Microbiology and Molecular Genetics, Oklahoma State University, Stillwater, Oklahoma, United States of America; DUMC, UNITED STATES

## Abstract

*Chlamydia trachomatis* is an obligate intracellular bacterium that replicates within a vacuole termed an inclusion. At the end of their intracellular developmental cycle, chlamydiae are released either by lysis of the host cell or extrusion of the intact inclusion. The inclusion membrane is extensively modified by the insertion of type III secreted inclusion membrane proteins, Incs, which contribute to inclusion membrane structure and facilitate host-pathogen interactions. An interaction was identified between the inclusion membrane protein, MrcA, and the Ca^2+^ channel inositol-1,4,5-trisphosphate receptor, type 3 (ITPR3). ITPR3 was recruited and localized to active Src-family-kinase rich microdomains on the inclusion membrane as was the Ca^2+^ sensor, STIM1. Disruption of MrcA by directed mutagenesis resulted in loss of ITPR3 recruitment and simultaneous reduction of chlamydial release by extrusion. Complementation of MrcA restored ITPR3 recruitment and extrusion. Inhibition of extrusion was also observed following siRNA depletion of host ITPR3 or STIM1. Chlamydial extrusion was also inhibited by the calcium chelator BAPTA-AM. Each of these treatments resulted in a concomitant reduction in phosphorylation of the myosin regulatory light chain (MLC2) and a loss of myosin motor activity at the end of the developmental cycle which is consistent with the reduced extrusion formation. These studies suggest that Ca^2+^ signaling pathways play an important role in regulation of release mechanisms by *C*. *trachomatis*.

## Author summary

At the end of their intracellular developmental cycle, chlamydiae exit host cells by one of two mechanisms; lysis of the host cell or extrusion of a membrane bound vesicle containing viable chlamydiae. Sequestration of infectious organisms within a vesicle is believed to aid in dissemination of the bacteria from sites of inflammation. Extrusion is known to be an active process dependent upon actin and the myosin motor complex yet the regulatory factors determining release by lysis or extrusion are poorly understood. Here we show that specific chlamydial inclusion membrane proteins form a complex with cellular calcium channels and sensors at unique sites on the chlamydial inclusion membrane where they influence activity of the myosin motor complex and extrusion.

## Introduction

*Chlamydia trachomatis* is a Gram-negative obligate intracellular bacterium that causes a variety of human and veterinary infections. Distinct serological variants, or serovars, are responsible for different diseases such as trachoma, the leading cause of infectious blindness worldwide (serovars A-C) [1], sexually transmitted diseases (serovars D-K) or a systemic disease referred to as lymphogranuloma venereum (serovars L1-L3) [2]. All chlamydiae are characterized by a biphasic development cycle that occurs exclusively within the confines of a membrane bound compartment of the host cell, the inclusion. The bacteria alternate between metabolically inactive infectious forms called elementary bodies (EBs) and metabolically active but noninfectious forms known as reticulate bodies (RBs) [3–5]. Soon after uptake, EBs differentiate into RBs which replicate by binary fission until the inclusion occupies a large part of the host cell. Beginning at about 18 h post-infection, a proportion of RBs begin differentiation back to EBs. At the end of the development cycle, EBs are released from the host cell either by lysis of the host cell or extrusion of the intact inclusion to initiate new cycles of infection [6].

Chlamydiae encode a type III secretion system (T3SS), which is a macromolecular complex used by Gram-negative bacteria to translocate bacterial effector proteins across host membranes [7, 8]. Among these secreted effectors are a number of inclusion membrane proteins (Incs) that are incorporated into the inclusion membrane to stabilize the membrane and mediate crucial host-pathogen interactions. Incs are characterized by a bilobed hydrophobic domain of 40 or more amino acids [9–11]. The number of Incs in *C*. *trachomatis* is predicted to be between 36–59 but varies in other chlamydial species [12–14]. These Incs represent a highly diverse set of proteins that are suspected of involvement in modulating host signaling pathways and cellular functions. The functions of several Incs have been elucidated [15–20] and presumptive interacting partners have been identified [21]. However, functional interactions for the majority of Incs remains to be defined and some appear to have structural roles in inclusion membrane stability [22, 23]. Certain of the Incs (IncB, IncC, CT101, CT222, CT223, CT224, CT228, CT288, and CT850) [24, 25] are enriched in specialized microdomains of the chlamydial inclusion membrane that are also enriched in active Src-family kinases and cholesterol that are believed to be platforms for interactions with the cytoskeleton [24].

In the initial description of the phenomenon, myosin was found to be important for the extrusion of *C*. *trachomatis* inclusions [6]. Recently, one of the *C*. *trachomatis* microdomain Incs, CT228, was found to play a role in control of the extrusion process via recruitment of myosin phosphatase target subunit 1 (MYPT1) to microdomains [20]. MYPT1 is part of myosin phosphatase that serves to regulate activity of the myosin motor by dephosphorylation of the myosin regulatory light chain, MLC2, which must be in a phosphorylated state for motor activity [26]. Very late in the *C*. *trachomatis* developmental cycle there is an overall dephosphorylation of MYPT1 in infected cells, however, there is an enrichment of phosphorylated MYPT1 at microdomains. This focal enrichment of inactive myosin phosphatase, serves to favor maintenance of phosphorylated, active MLC2 and myosin light chain kinase (MLCK) which promote activity of the myosin motor complex and extrusion of the inclusion [20]. How the activity of MYPT1 itself is regulated remains unknown. The presence of multiple Incs at the microdomain suggests that any regulation may be difficult to resolve. Cellular signals potentially regulating activity of this complex are not known.

In the present study, we found that type 3 inositol-1,4,5-trisphosphate receptor (ITPR3), a calcium channel, is recruited to the *C*. *trachomatis* inclusion through an interaction with the inclusion membrane protein, MrcA (annotated as CT101 in *C*. *trachomatis* serovar D and CTL0356 in serovar L2). MrcA and ITPR3 co-localize with active pY419-Src-family kinase at inclusion membrane microdomains. Knockout of MrcA in *C*. *trrachomatis* or depletion of cellular ITPR3 by siRNA interference resulted in decreased extrusion by *C*. *trachomatis*. Similar results were obtained when *C*. *trachomatis* infected cells were treated with the calcium chelator BAPTA-AM. Inhibition of extrusion correlated with reduction of phosphorylated myosin light chain (pS19-MLC2) which is essential for myosin motor activity. Altogether the results suggest that MrcA functions in recruitment of the inositol 1,4,5-triphosphate receptor, type 3 (ITPR3) into a regulatory complex comprised of both chlamydial and host proteins. We have therefore named the inclusion membrane protein CT101 Myosin Regulatory Complex subunit A (MrcA). Regulation of chlamydial exit strategies at the end of the developmental cycle appears to involve calcium signaling as an upstream regulator of a phosphoregulatory pathway which determines the relative rates of *C*. *trachomatis* exit by lysis or extrusion.

## Results

### ITPR3 is recruited to and interacts with MrcA within inclusion microdomains

The chlamydial inclusion membrane exhibits localized structures, termed microdomains, that are enriched in host active Src-family kinases and cholesterol [24]. Several inclusion membrane proteins are enriched in these microdomains including MrcA, CT850, CT222, IncB, CT223, CT224, CT228, IncC and CT288 [24, 25].

**Fig 1:**
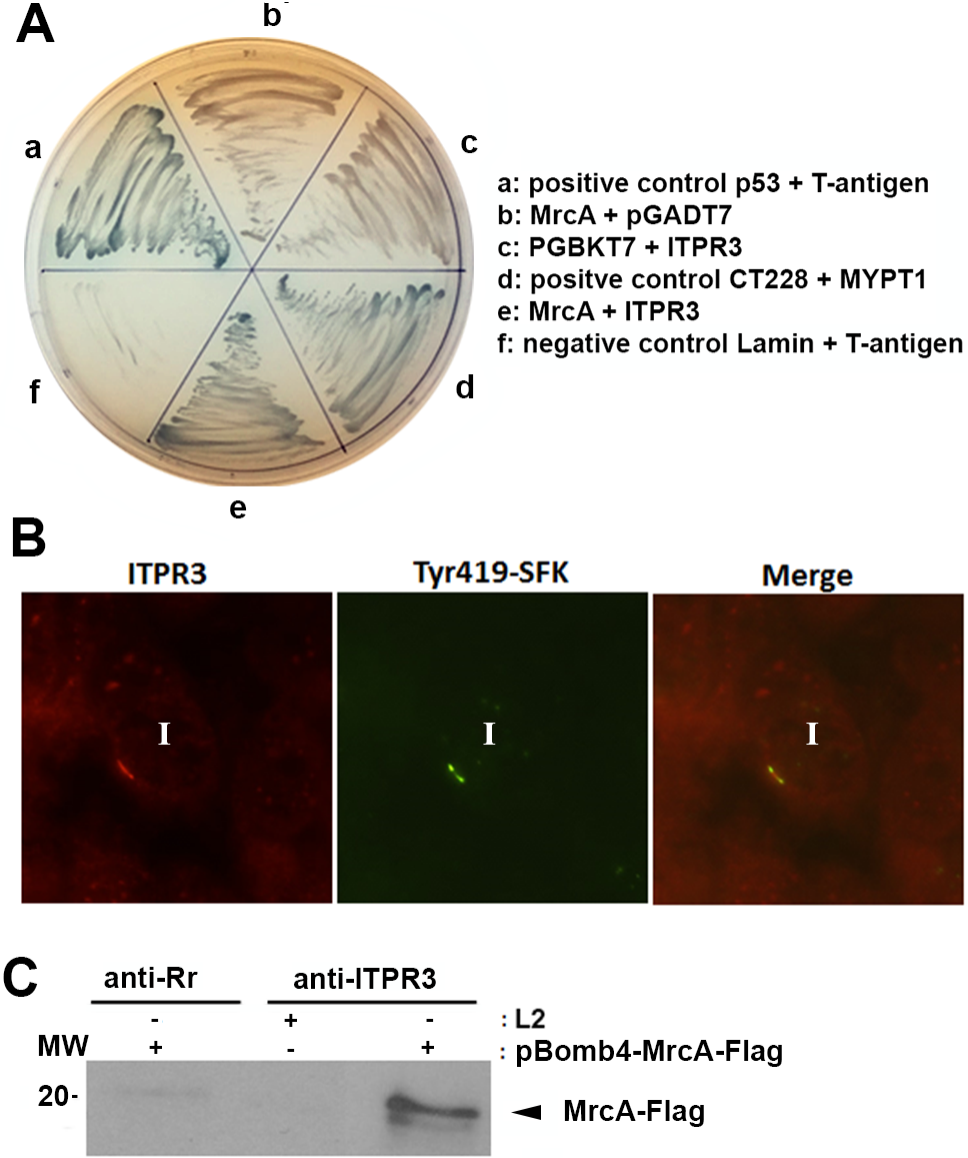
Interaction of MrcA with ITPR3. (A) Yeast-2-hybrid identification of ITPR3 as a binding partner of MrcA. Interactions in the yeast-two hybrid were verified by screening diploids grown on SD-Trp Leu and high stringency SD-Trp Leu His Ade + α Gal. Interactions were detected between the positive controls p53 + T-antigen (a) and CT228 and MYPT1 (d) as well as MrcA and ITPR3 (e) as evidenced by α-Galactosidase activity on Trp Leu His Ade resulting in blue yeast colonies. No interactions were detected with MrcA and pGADT7 (b), pGBKT7 and ITPR3 (c) and the negative control Lamin + T antigen (f). (B) ITPR3 is recruited to the inclusion and colocalizes with active kinase. Hela cells were infected with *C*. *trachomatis* L2. 24 hrs post infection cells were fixed and immunolabeled with anti-active Src-family kinase (Tyr419) (green) and anti-ITPR3 (red). Specimens were examined by confocal microscopy. ITPR3 shows discrete microdomain-like staining patterns that colocalize with active Src kinase. Inclusions are indicated by “I”. Bar = 10 μm. (C) ITPR3 interacts with MrcA. T150 flasks of Hela cells were infected with *C*. *trachomatis* transformed with pBomb4- MrcA -Flag or parental *C*. *trachomatis* L2. 48 hrs post infection cells were harvested and lysate prepared for co-immunoprecipitation. The supernatant from infected lysate was added to Protein-A beads that had been pre-loaded with anti-ITPR3 or with anti-*R*. *rickettsii* antibodies (anti-Rr). Immunoblots were probed with anti-Flag.

Yeast-two hybrid screening was used to identify potential interacting partners for MrcA. The C- terminus of MrcA (amino acids 55–153) was cloned into pGBKT7 (Clontech), sequenced, and transformed into yeast strain AH109. AH109 carrying pGBKT7-MrcA was used as bait to screen a HeLa cDNA library in the yeast strain Y187. Interacting diploids were first identified in a low stringency screen and then confirmed under higher stringency conditions. Prey plasmid DNA from positive interacting partners was isolated and sequenced. Three of the interacting positive diploids sequenced were found to be identical prey plasmids carrying the C-terminus of the inositol 1,4,5-triphosphate receptor type 3 (ITPR3) gene fused to Gal4 AD [position 6036 bp; 2045 aa of ITPR3]. Bait dependency experiments verified the interaction between MrcA and ITPR3 as the diploid was able to grow and express α-galactosidase in high stringency conditions identical to the two positive controls p53 + T-antigen and CT228 and MYPT1 (Fig 1A). ITPRs are calcium channels that mediate the release of Ca^2+^ from intracellular stores in response to binding of IP3 [27]. Immunofluorescence microscopy revealed that ITPR3 was recruited to and co-localized with active Src-family kinases in inclusion microdomains (Fig 1B).

A shuttle vector system, pBOMB4, was used to express MrcA fused with a C-terminal Flag tag [28]. *C*. *trachomatis* transformed with pBOMB4 or pBOMB4 containing MrcA-Flag had similar growth rates to wild-type L2 (S1 Fig). MrcA-Flag induced with tetracycline was localized in inclusion microdomains (S1 Fig). To confirm the interaction between MrcA and ITPR3, coimmunoprecipitation was performed. MrcA-flag was coprecipitated by anti-ITPR3 from lysates of cells infected with *C*. *trachomatis* L2 transformed with pBOMB4-MrcA-Flag but not from lysates of cells infected with parental *C*. *trachomatis* L2 nor by precipitation with an irrelevant antiserum (Fig 1C).

### Disruption of MrcA or ITPR3 depletion affect chlamydial release by extrusion

**Fig 2:**
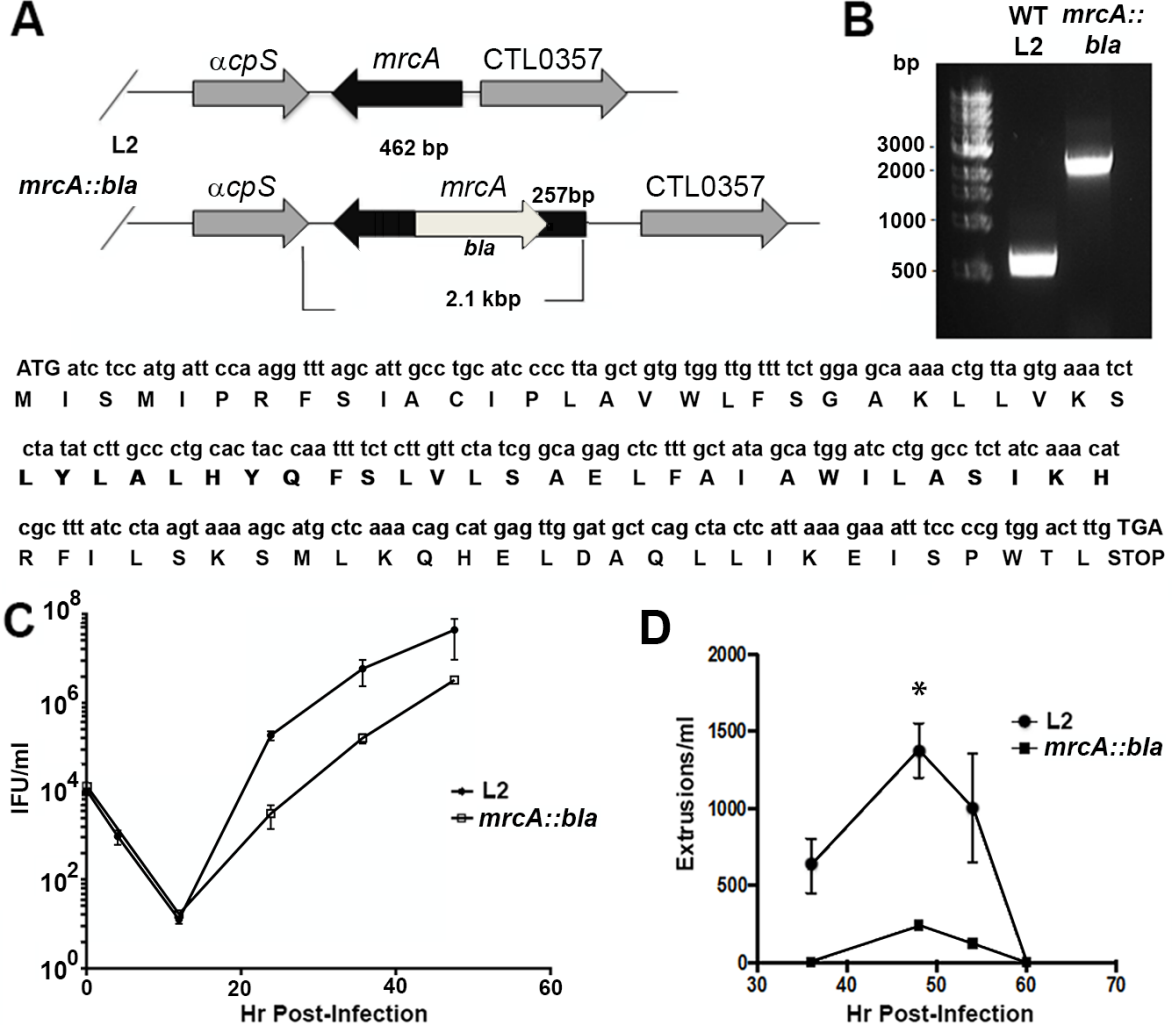
Generation of an *mrcA::bla* mutant. (A) Sanger sequencing of isolated DNA using gene-specific primers was conducted to verify orientation and insertion of the intron. (B) PCR products were separated on a 1% agarose gel, DNA was visualized using ethidium bromide staining. WT, Wild type. (C) Hela cells infected with wild-type L2 or *mrcA::bla* L2 were incubated for 0 h, 4 h, 12 h, 24 h, 36 h, or 48h. Cells were lysed in water and replated on fresh monolayers to enumerate the progeny IFUs. Experiments were performed in triplicate (n = 3, error bars indicate SEM. (D) Hela cells infected with wild-type L2 or *mrcA::bla* L2 at an MOI = 1. At various time points post infection, the supernatants were removed and gently pelleted by centrifugation at 1200rpm. The resulting pellet was resuspended in 100 μl of media. Extruded inclusions free of host cell nuclei were enumerated (n = 3, error bars represent SEM). The difference was significant (P<0.01) by an unpaired Students T-test at 48hpi.

The TargeTron pACD4K vector was modified for use in *C*. *trachomatis* [25] and used to disrupt *mrcA* (Fig 2A). To verify disruption of *mrcA*, we amplified genomic DNA isolated from wild-type L2 and from the L2 *mrcA::bla* mutant using gene specific primers. Agarose gel electrophoresis of the PCR product indicated insertion of the group II intron by the addition of 1.7 kB in the mutant compared to the wild-type (Fig 2B). DNA sequencing of *mrcA::bla* verified insertion of the intron in the antisense orientation relative to *mrcA* (Fig 2A). The disruption of MrcA slightly but significantly reduced the growth rate (Fig 2C).

We determined extrusion formation in the *mrcA::bla* mutant strain. Hela cells were infected with *C*. *trachomatis* wild-type L2 or *mrcA::bla* with an MOI of 1. At different time points post-infection extrusions from an equal number of infected cells were enumerated. A peak of extrusion formation was observed at 48 hrs post infection. The disruption of MrcA significantly inhibited extrusion formation compared to the wild-type strain (Fig 2D). Plaque formation on Vero cells, however, was not disrupted by this mutation.

**Fig 3:**
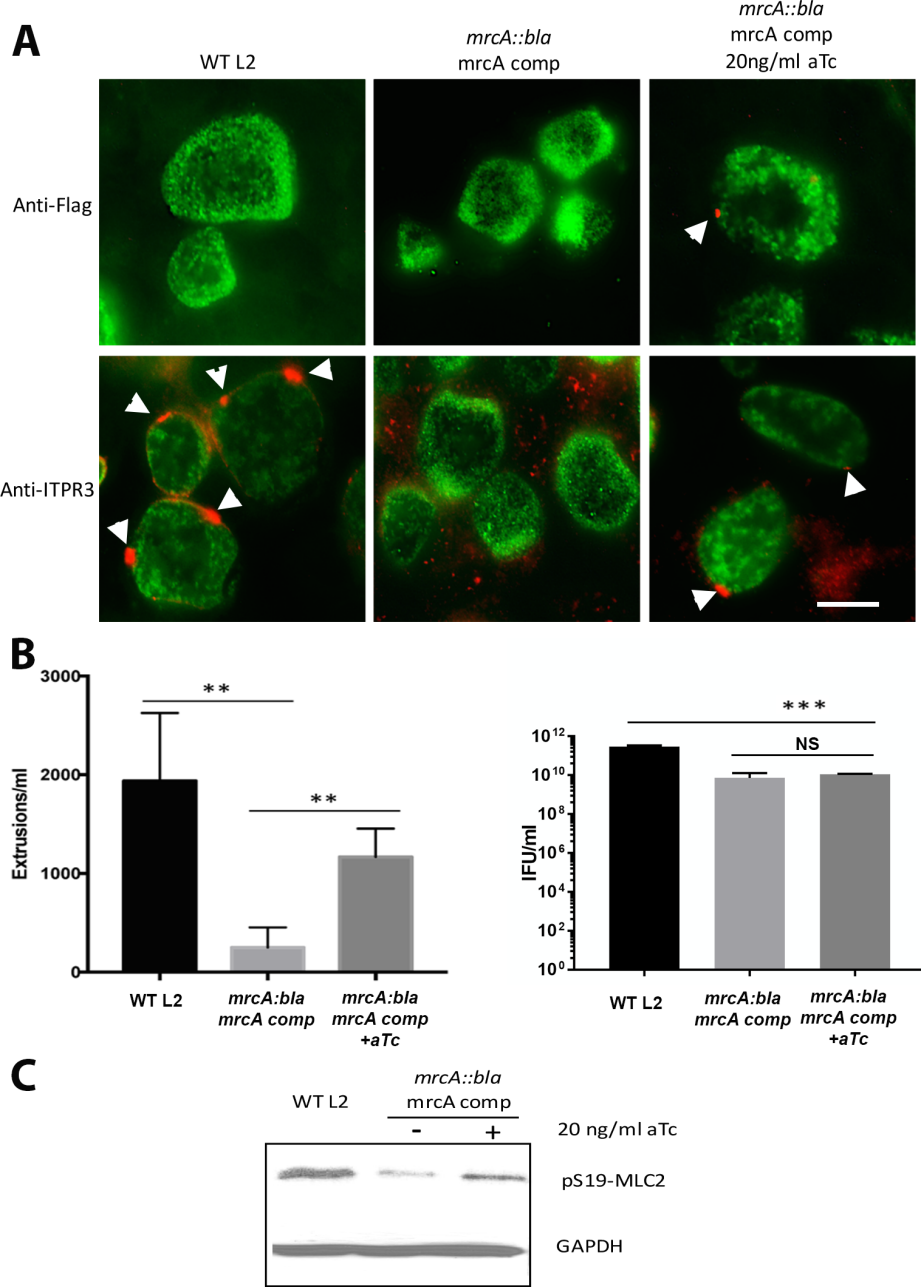
Complementation of *mrcA::bla* L2. Hela cells were infected at an MOI of 1 with the *mrcA::bla* mutant strain and the pBomb3 MrcA complemented strain either uninduced or induced with aTC. (A) at 24 hrs post-infection, cells were fixed with methanol and probed with anti-Flag (red) or anti-ITPR3 (red), both of which localize to microdomains, and anti-MOMP (green) antibodies to identify the chlamydiae. Arrowheads indicate Flag or ITPR3, respectively, localized at microdomains on the inclusion membrane. Bar = 10 μm. (B) Free extrusions were enumerated at 48 hrs post-infection. (n = 3, error bars represent the SEM, the difference was significant at P<0.0001;****) by an unpaired Students T-test. Right hand panel indicates infectious progeny EB formation (IFU/ml) (n = 3, error bars represent the SEM, the differences between the parental strain and the *mrcA::bla* mutant strain or complemented *mrcA::bla* strain were significant at P<0.0005;***) by an unpaired Students T-test. The difference between the *mrcA::bla* strain and the complemented *mrcA::bla* strain was not significant (ns). (C) Immunoblot showing levels of pS19-MLC2 at 44 hrs post-infection, and GAPDH as a loading control.

To confirm a role for MrcA in recruitment of ITPR3 and extrusion formation, we complemented *mrcA::bla* with Flag-tagged MrcA from a tetracycline-inducible promoter using the pBOMB3 vector [29]. The *mrcA::bla* strain did not recruit ITPR3 to the inclusion membrane (Fig 3A). Quantitation of ITPR3 recruitment indicated 85.5% +/- 1.2% ITPR3 positive microdomains on parental *C*. *trachomatis* but only 4.1% +/- 0.3% on the *mrcA::bla* mutant (Mean +/- SEM, n = 3, p<0.0001). Induction of MrcA expression by 20 ng/ml aTc restored MrcA expression and appropriate localization to microdomains on the inclusion membrane. ITPR3 recruitment to microdomains was also recovered with induction of MrcA expression (Fig 3A). Extrusion formation was significantly rescued in the complemented strain although the growth defect in the *mrcA::bla* strain was not restored (Fig 3B). Phosphorylation of MLC2 also increased in the complemented *mrcA::bla* mutant relative to the uninduced strain (Fig 3C).

**Fig 4:**
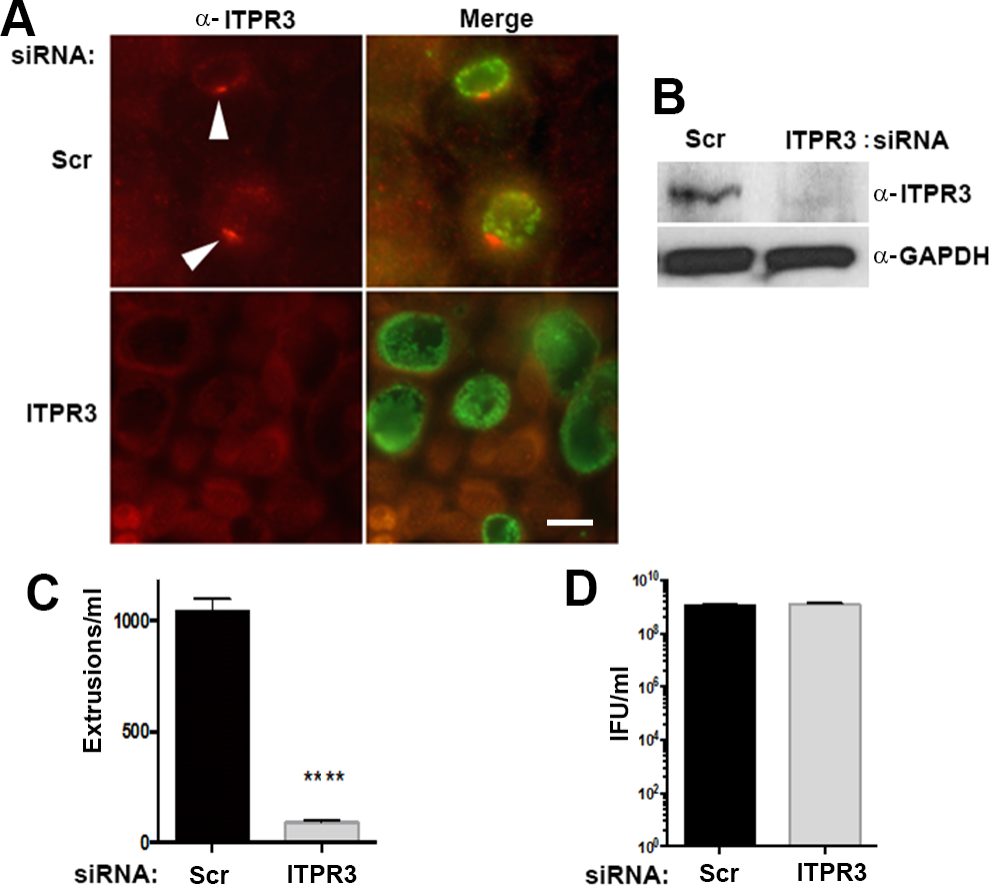
Depletion of ITPR3 inhibits extrusion formation. Hela cells were treated with siRNA corresponding to ITPR3 or a scrambled negative control siRNA (Scr) for 72 hours then infected with *C*. *trachomatis* wild-type L2. (A) At 24 hr post-infection, cells were fixed and stained with anti-ITPR3 (red) (Arrowheads) and anti-MOMP (green) to verify the depletion of ITPR3. Bar = 10 μm. (B) Immunoblots confirmed knock out of ITPR3. (C) Effect on extrusion formation by siRNA depletion of the ITPR3 by siRNA. (D) Effect on total infectious progeny production by siRNA depletion of the ITPR3. Experiments were performed in triplicate (n = 3; error bars represent SEM). The difference was significant (P<0.0001; ****) by an unpaired Students T-test.

The effects of siRNA depletion of ITPR3 on chlamydial release by extrusion were also assessed. Efficacy of siRNA knockdown was confirmed by immunofluorescence (Fig 4A) and by immunoblot (Fig 4B). At 48 hrs post- infection, ITPR3 depletion significantly reduced extrusion formation but had no effect on total progeny IFUs (Fig 4C and 4D). Collectively, the results demonstrate that ITPR3 must be present and appropriately localized to function in this process.

### Extrusion inhibition correlates with diminution of pT853-MYPT1 and pS19-MLC2 recruitment

**Fig 5:**
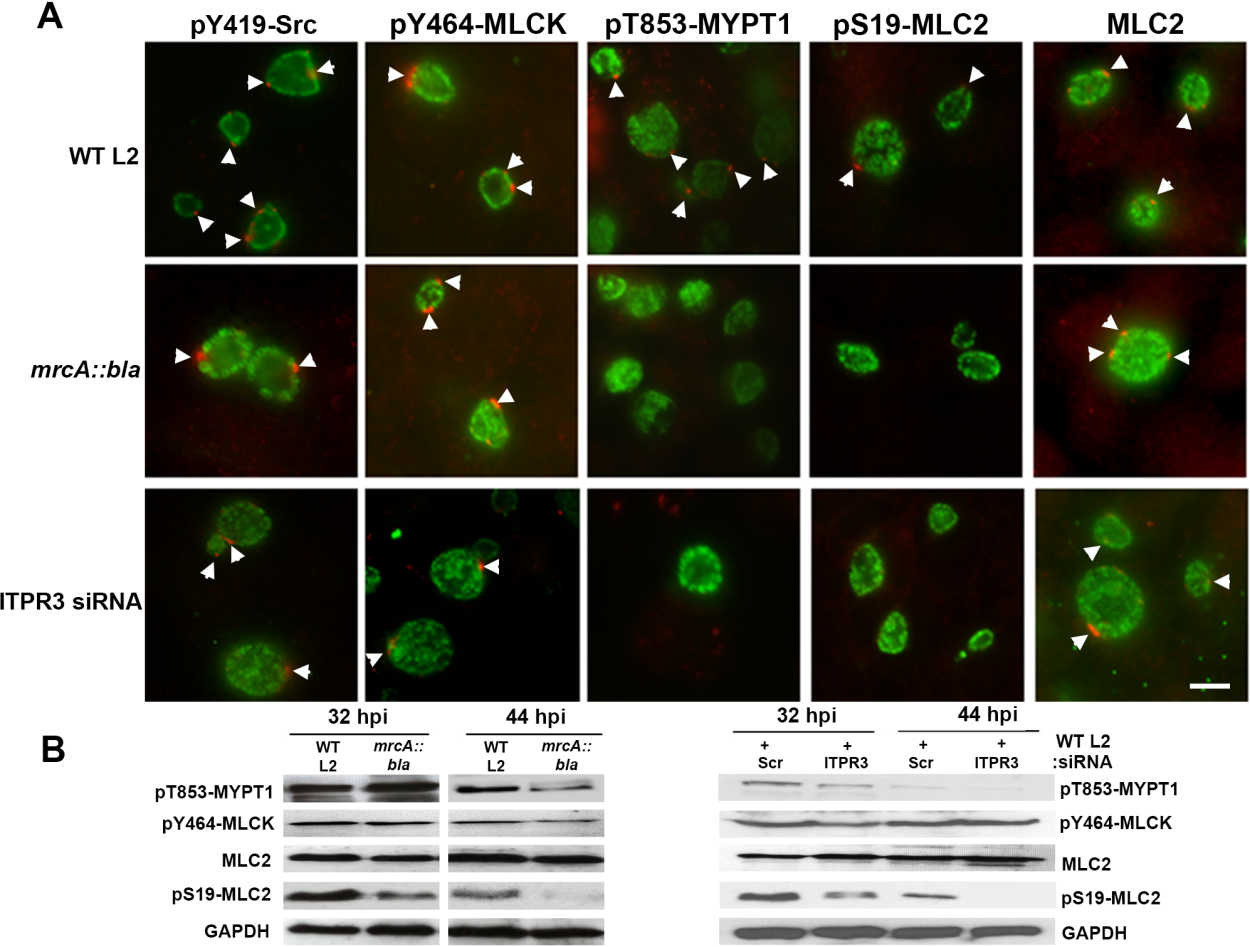
MrcA disruption or ITPR3 depletion decreases pT853-MYPT1 and pS19-MLC2 recruitment to chlamydial inclusion. (A) Hela cells were infected with *C*. *trachomatis* L2 or *mrcA::bla* L2. ITPR3 was depleted from Hela cells using siRNA and infected with *C*. *trachomatis* L2. At 24 hrs post infection, cells were fixed and stained with anti-pY419-Src (red), anti-pY464-MLCK (red), anti-pT853-MYPT1 (red), anti-pS19-MLC2 (red), anti-MLC2 (red) (all red staining of microdomains is indicated by arrowheads), and anti-MOMP (green). Bar = 10 μm. (B) Immunoblots of L2 and *mrcA::bla* infected HeLa cells or ITPR3-depleted, L2 infected HeLa cells probed with anti-pT853-MYPT1, anti-pY464-MLCK, anti-MLC2, and anti-pS19-MLC2, with anti-GAPDH as a loading control.

We investigated whether the reduction in extrusion caused by MrcA knockout or by ITPR3 depletion was due to changes in the myosin phosphoregulatory pathway [20]. Phosphorylated MLC2 along with myosin IIA and IIB form the active myosin motor complex [26]. Myosin phosphatase (MYPT1) dephosphorylates pS19-MLC2 and thus inhibits myosin motor activity and chlamydial extrusion [20]. When phosphorylated at T695 and T853, the phosphatase activity of MYPT1 is inhibited and can no longer dephosphorylate pS19-MLC2. Late in *C*. *trachomatis* infection there is an overall reduction in phosphorylated, inactive MYPT1 but what remains is concentrated at microdomains and serves to focus myosin motor activity on the inclusion [20]. We did not observe a reduction in recruitment of pY419-Src, active tyrosine-464 phosphorylated Ca^2+^/Calmodulin-dependent myosin light chain kinase (pY464-MLCK), or MLC2 to microdomains in *mrcA::bla* as compared with wild-type L2, but there was a notable reduction of pT853-MYPT1 and pS19-MLC2 recruitment (Fig 5A). Levels of pY464-MLCK were equivalent in wild type and mutant strains (Fig 5B). A reduction in levels of pT853-MYPT1 is typical [20] and was verified by immunoblotting (Fig 5B). Importantly, cellular levels of pS19-MLC2 were dramatically reduced in the *mrcA::bla* mutant strain at 44 hr post-infection, consistent with a loss of myosin motor activity. We observed similar results with knockdown of ITPR3 in that there was reduced pT853-MYPT1 and pS19-MLC2 recruitment to the chlamydial inclusion (Fig 5A) and near absence of pS19-MLC2 in immunoblots (Fig 5B). The decreased rates of extrusion formation upon disruption of MrcA or ITPR3 thus correlate with the reduction of pT853-MYPT1 and pS19-MLC2 recruited to the inclusion membrane.

### Recruitment of the ER calcium sensor, STIM1, to microdomains

**Fig 6:**
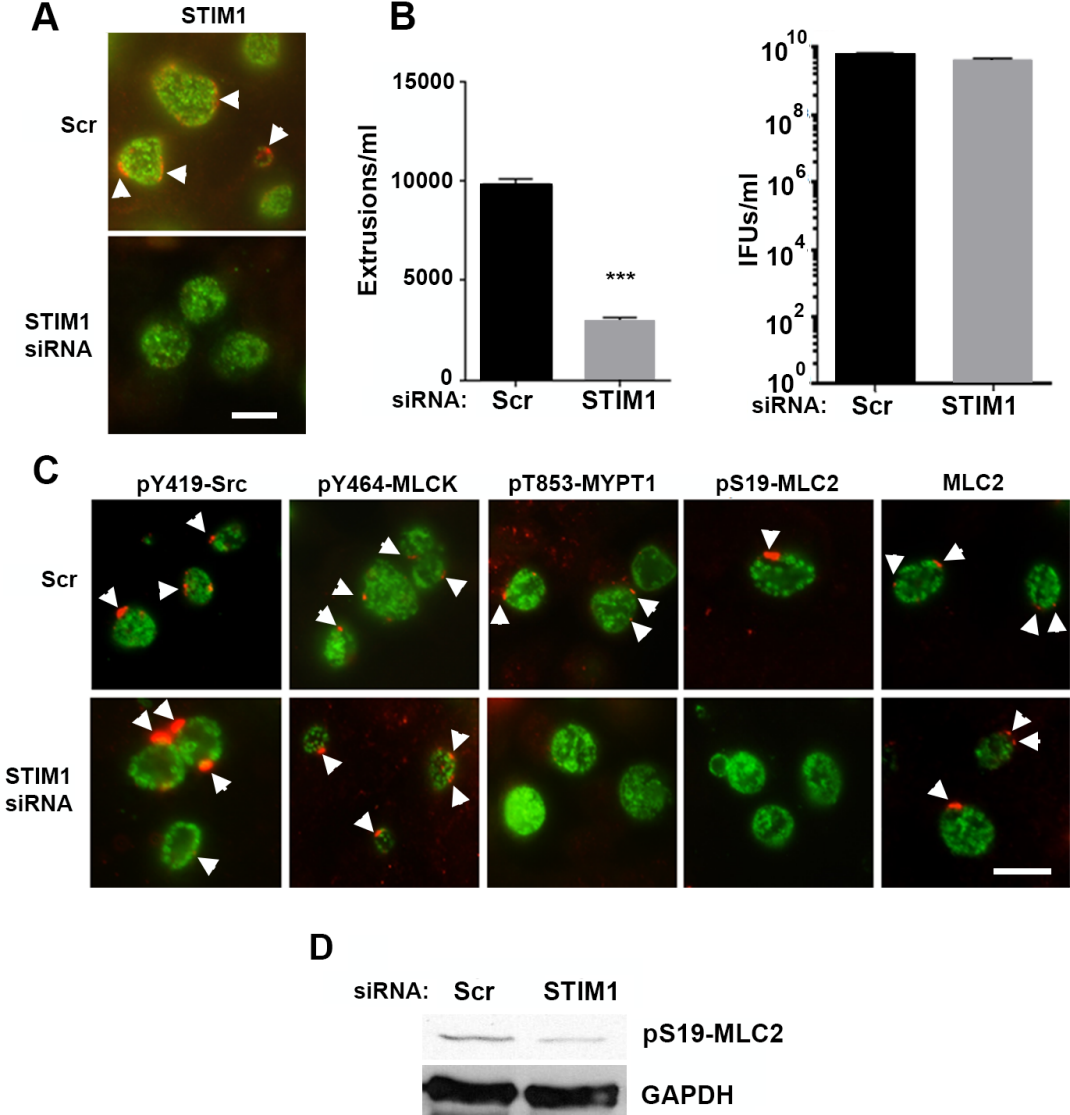
Effect of STIM1 depletion on extrusion formation and recruitment of pT853-MYPT1 and pS19-MLC2 to the chlamydial inclusion. STIM1 was depleted from HeLa cells using siRNA and infected with *C*. *trachomatis* L2. (A) STIM1 depletion was confirmed by immunofluorescence on *C*. *trachomatis*-infected cells using an anti-STIM1 antibody (red) (arrowheads). (B) Extrusions were significantly reduced (n = 3; error bars indicate SEM, the difference was significant at P < 0.0001; ****) by an unpaired Students T-test at 48 hrs post-infection with no significant reduction in progeny formation. (C). At 24 hrs post infection, HeLa cells transfected with siRNA against STIM1 or a scrambled control siRNA (Scr) were fixed and stained with anti-pY419-SFK (red), anti-pY464-MLCK (red), anti-pT853-MYPT1 (red), anti-pS19-MLC2 (red), anti-MLC2 (red) (all red labeling of microdomains is indicated by arrowheads), and anti-MOMP (green). Bar = 10 μm. (D) Immunoblot showing reduction in STIM1 in siRNA treated cells.

Recently, the stromal interaction molecule 1 (STIM1) was shown to be recruited to *C*. *trachomatis* inclusion membrane contact sites (MCS) [30]. STIM1 is a ER resident Ca^2+^ sensor that detects ER Ca^2+^ store depletion and acts, in concert with ORAI1, to trigger Ca^2+^ influx into cells [31]. We investigated the recruitment of STIM1 to microdomains and found that STIM1 colocalized with both MrcA and pY464-MLCK (S2 Fig). Depletion of STIM1 by siRNA (Fig 6A) led to a significant decrease in number of extrusions observed at 48 hrs (Fig 6B) but had no effect on chlamydial replication. As observed with depletion of ITPR3, the reduction in extrusion formation was correlated with reduced pT853-MYPT1 and pS19-MLC2 recruitment to the chlamydial inclusion (Fig 6C) coupled with a decrease in phosphorylation of MLC2 (Fig 6D).

### Intracellular calcium chelation by BAPTA-AM reduces chlamydial extrusion

**Fig 7:**
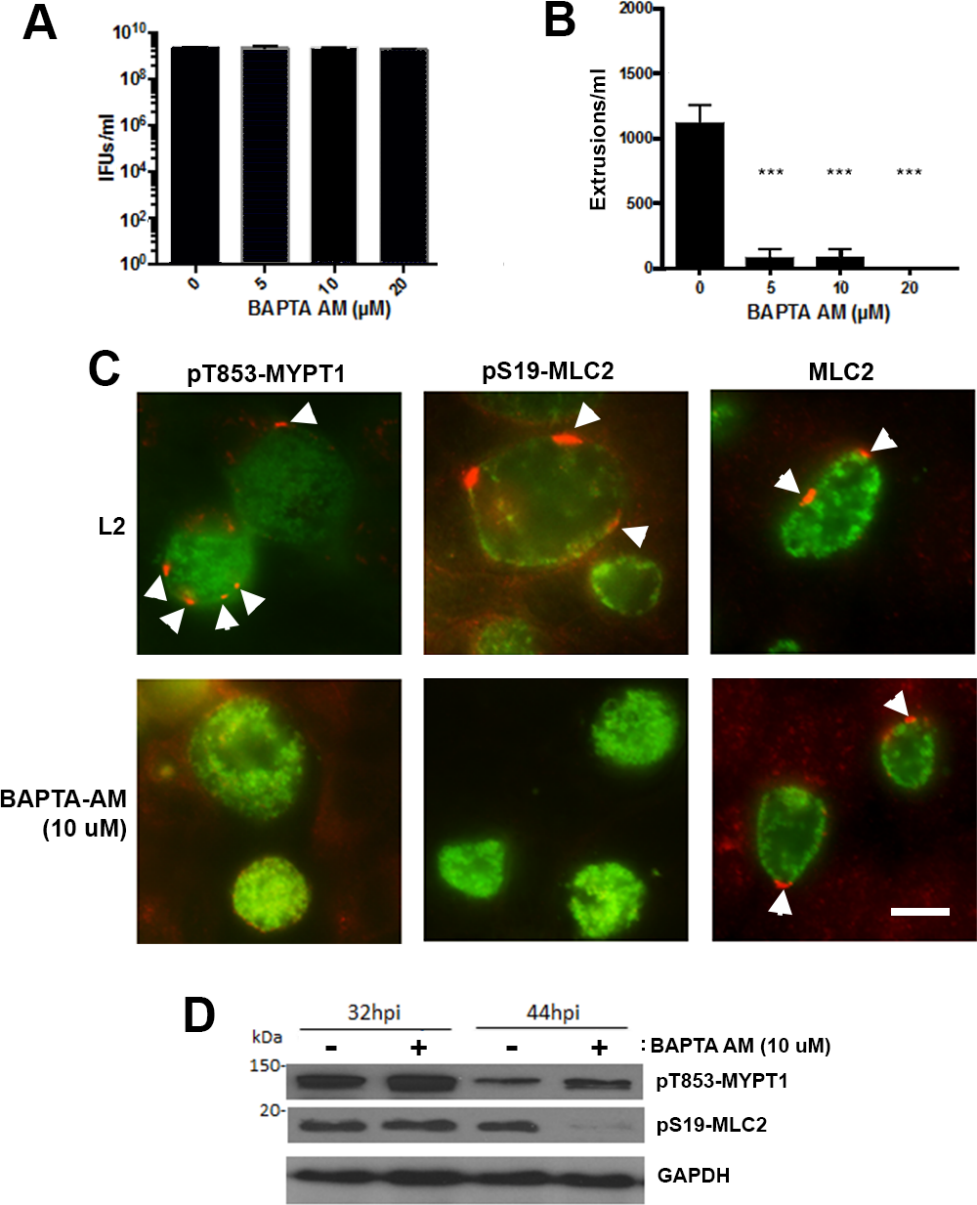
Effects of BAPTA-AM, an intracellular calcium chelator, on extrusions. Hela cells were infected with wild-type L2 at an MOI = 1. At 24 hrs post infection, media was removed and replaced with calcium-containing (L2) or calcium-free media in which various amounts (0, 5, 10, 20 μM) of BAPTA-AM was added. (A) Cells were lysed in water and replated on fresh Hela cells monolayers to enumerate the IFU; (n = 3, error bars represent SEM). The difference was not significant. (B) Free extrusions were enumerated at 48 hrs post infection (n = 3, error bars represent SEM, the difference was significant at P < 0.0001; ****) by an unpaired Students T-test. (C) Indirect immunofluorescence, *C*. *trachomatis* L2 infected cells with or without treatment of BAPTA-AM (10 μM) were fixed and stained with anti-pT853-MYPT1 (red) (arrowheads), anti-pS19-MLC2 (red) (arrowheads) and anti-MOMP (green). Bar = 10 μm. (D) Immunoblot analysis of cells infected with wild-type L2 with treatment of BAPTA-AM for 32 and 44 hrs post-infection showing levels of pT853-MYPT1, pS19-MLC2, and GAPDH as a loading control.

Both the calcium channel, ITPR3, and the Ca^2+^ sensor, STIM1, are important for extrusion formation, suggesting a role for Ca^2+^ signaling in regulation of extrusion. The role of intracellular Ca^2+^ signaling in the extrusion process was therefore examined. *C*. *trachomatis*-infected cells at 24 hrs were incubated in calcium-free medium supplemented with various concentrations (0, 5, 10, or 20 μM) of the calcium chelator 1,2-bis (2-aminophenoxy) ethane-N,N,N’,N’ tetraacetate-acetoxymethyl ester (BAPTA-AM). Extrusion formation at 48 hrs post infection were completely inhibited, however, BAPTA-AM did not affect infectious progeny formation (Fig 7A and 7B). Similar to the results shown above, we observed reduction of recruitment of phosphorylated MYPT1 and MLC2 into inclusion microdomains (Fig 7C). However, immunoblots showed that BAPTA-AM treatment increased total cellular pT853-MYPT1 levels after 32 hrs or 44 hrs treatment. Again, inhibition of Ca^2+^ signaling significantly impaired phosphorylation of MLC2 at 44 hrs post infection (Fig 7D).

To further examine the Ca^2+^ release function of ITPR3 in extrusion formation, we treated infected Hela cells with caffeine which is known to inhibit ITPR mediated Ca^2+^ responses in a subtype 3 specific manner [32]. Caffeine significantly reduced the extrusion formation but had no effect on total IFUs (S3 Fig).

## Discussion

**Fig 8:**
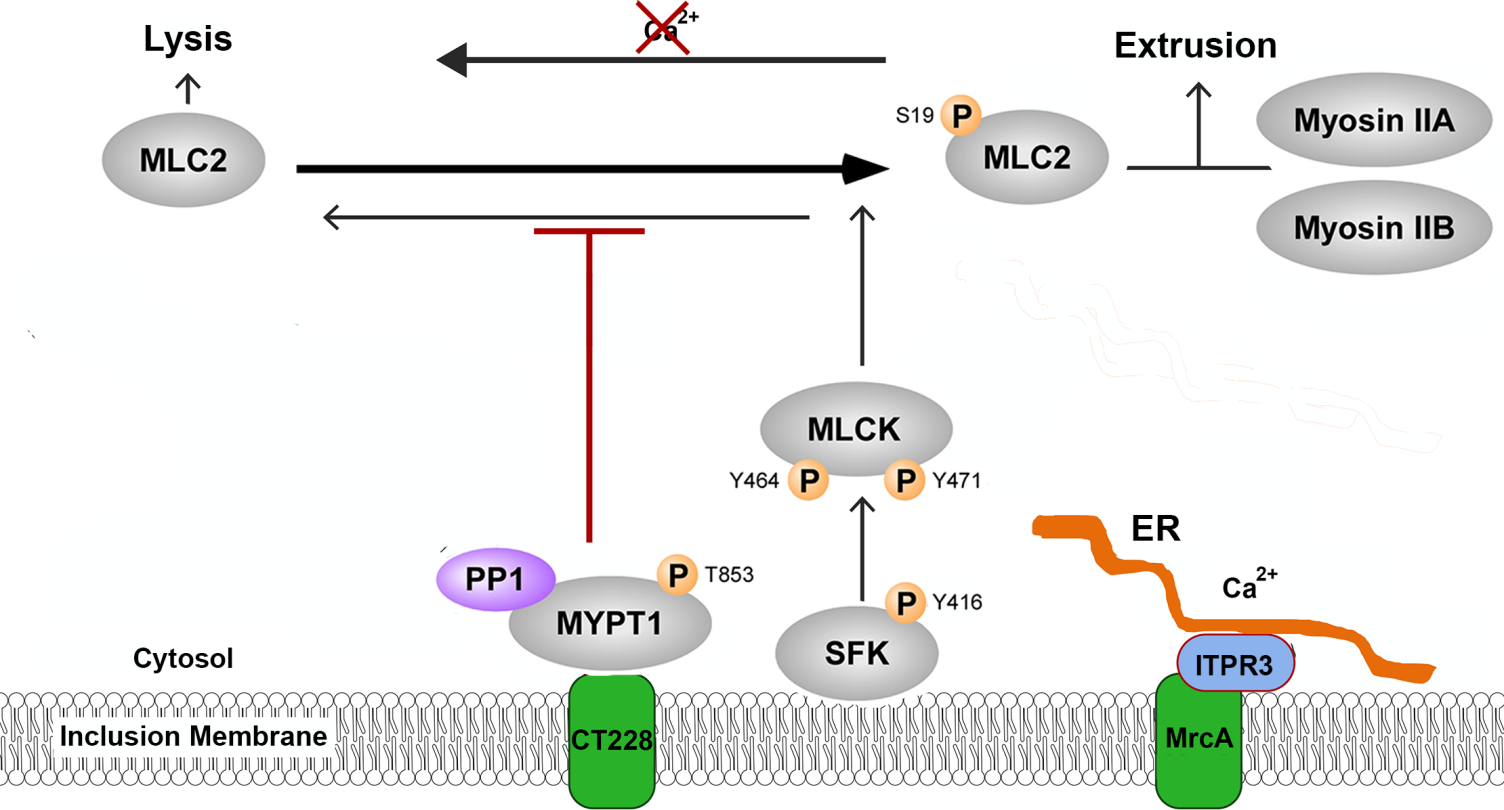
Schematic representation of myosin complex pathway in *C*. *trachomatis* microdomains. The myosin phosphatase pathway in *C*. *trachomatis* microdomains as described [20]. The phosphorylation state of MLC2 regulating the balance of exit by lysis and extrusion, is determined by kinase activity of MLCK and phosphatase activity of MYPT1. Through interaction with MrcA, ITPR3 is recruited to inclusion-ER contact sites or microdomains where it is expected to modulate local Ca^2+^ concentrations which influence the relative activities of both myosin kinase and myosin phosphatase to promote extrusion.

*C*. *trachomatis* has evolved at least two distinct mechanisms of host cell escape, cell lysis and extrusion [6, 33]. Lysis consists of sequential rupture of the chlamydial inclusion membrane and cellular plasma membrane to release EBs into the extracellular environment thus results in death of the host cell. In contrast, extrusion represents a process in which membrane-bound inclusions containing viable EBs are released by a process resembling exocytosis. This process involves cytoskeletal activity including actin polymerization and the myosin motor complex [6]. A model in which key elements of the myosin phosphatase pathway regulate myosin motor activity and extrusion formation has been proposed [20] (Fig 8). In this model, activity of the myosin motor complex is focused at specific points on the inclusion membrane. The chlamydial inclusion membrane protein, CT228, is known to recruit the myosin phosphatase target subunit 1 (MYPT1) component of the myosin phosphatase complex to inclusion membrane microdomains, yet the mechanisms regulating the activity of myosin phosphatase had not been defined. Here we show that another Inc present in microdomains, MrcA, recruits ITPR3, an inositol 1,4,5-triphosphate responsive Ca^2+^ channel that functions in the regulation of multiple signaling pathways [27] and is essential to the extrusion process. STIM1, an ER resident Ca^2+^ sensor [31], is also recruited to these microdomains and required for extrusion. Indeed, Ca^2+^ appears critical to the extrusion process as depletion effectively inhibits extrusion. Collectively, the results indicate that *C*. *trachomatis* inclusion membrane microdomains are platforms for interactions with the cytoskeleton and that Ca^2+^ signaling is an essential component of extrusion activation.

The motor activity of myosin II is activated by phosphorylation of the myosin regulatory light chain (MLC2) and functions in multiple cellular contractile activities. Phosphorylation of MLC2 is controlled by the antagonistic activities of the Ca^2+^/Calmodulin-dependent myosin light chain kinase (MLCK) and myosin light chain phosphatase which phosphorylate and dephosphorylate MLC2, respectively [26]. The activities of the kinase and the phosphatase are themselves regulated by phosphorylation state. Myosin light chain kinase is active when phosphorylated at tyrosine position 464 or 471 and myosin phosphatase is inactivated by phosphorylation of the MYPT1 subunit at threonine 695 or 853. Late in *C*. *trachomatis* infection there is an overall depletion of cellular phosphorylated MYPT1 yet there is a localized enrichment of pT-853-MYPT1 at microdomains on the inclusion membrane [20]. Because pT853-MYPT1 is the inactive form of the phosphatase that would otherwise dephosphorylate and therefore inactivate MLC2, the myosin motor complex remains active and focused at the microdomain. Disruption of MrcA or depletion of ITPR3 lead to a loss of the focal enrichment of pT-853-MYPT1 and pS19-MLCS at microdomains and greatly reduced levels of MLC2 late in the chlamydial developmental cycle which correlates with the reduction in extrusion formation. Calcium chelation precisely mimics these effects suggesting a role for calcium signaling in regulation of extrusion.

Inositol 1,4,5-triphosphate receptors release Ca^2+^ from intracellular stores in response to the simultaneous binding of both IP3 and Ca^2+^ [27]. ITPRs are localized to the ER where they form homo- or hetero-tetrameric channels, often in macromolecular signaling complexes whose specificity is conferred by association with a variety of proteins involved in different signaling pathways [27]. These associations occur in complexes or microdomains at the plasma membrane or ER-membrane junctions with cellular organelles and serve to spatially and temporally coordinate Ca^2+^ signaling within cells [34]. The Stromal Interaction Molecule 1 (STIM1) has also been recently described as recruited to microdomains/MCS [30] although function was not defined. STIM1 is an ER-localized Ca^2+^ sensor that, upon depletion of ER Ca^2+^, translocates to the plasma membrane to interact with Orai1 and activate store-operated calcium entry (SOCE) [31, 35, 36]. We confirmed the association of STIM1 with microdomains and found that it is also required for extrusion. STIM1 is known to form complexes with ITPRs [37]. Multiple Ca^2+^ signaling proteins thus are recruited and active as part of the extrusion process. Increased cytosolic Ca^2+^ can be expected to have a direct effect on several regulatory components of the myosin motor complex. MLCK is in an autoinhibited state but undergoes a conformational change in the presence of Ca^2+^/calmodulin such that it is activated to phosphorylate MLC2 [38]. Ca^2+^ is also known to activate ROCK [39], which phosphorylates MYPT1 [40] to down-regulate phosphatase activity. The combined effect of increased intracellular Ca^2+^ is activation of myosin light chain kinase and inhibition of the antagonistic myosin phosphatase to favor phosphorylation and thus activation of MLC2 and myosin motor activity promoting extrusion.

In one of the initial studies describing the extrusion process, calcium signaling was shown to be important for plasma membrane disruption during the lytic release but depletion of intracellular Ca^2+^ did not appreciably influence rates of extrusion [6]. In that study, Ca^2+^ depletion was accomplished by incubation with BAPTA-AM in Ringer's solution for 1 hr prior to analysis of extrusion. A technical difference in our procedure was incubation for 12 hr at 37^o^ C in Ca^2+^ free RPMI-10 plus BAPTA-AM. Under these conditions, extrusion formation was almost completely inhibited.

We previously described areas of the *C*. *trachomatis* inclusion membrane that are enriched in at least four inclusion membrane proteins, IncB, MrcA, CT222, and CT850, which we termed microdomains [24]. These microdomains are defined as being enriched in active Src-family kinases as shown by phosphorylation of Src at Tyr419 (pY-419). Each of the chlamydial or host proteins associated with microdomains were co-localized with active Src-family kinases. Based upon the interaction of CT850 with centrosomes and the dynein light chain (DNLT1), we proposed that these microdomains served as platforms for interactions with the cytoskeleton [24, 41]. Subsequently, localized enrichment of an ER resident protein, ceramide-ER-transferase (CERT) was noted on the inclusion membrane and these areas of ER association given the term membrane contact sites (MCS) [30]. The term pathogen synapse has also been applied to these structures [42]. Based upon co-staining with anti-pY416-Src and combinations of described components of these macromolecular complexes, we believe each of these terms describes the same structure. We have retained the term microdomain here for convenience and because the term is also used to describe regulatory complexes formed with ITP-receptors [43].

Other *C*. *trachomatis* Incs enriched in microdomains include IncC, CT223, CT224, CT228, and CT288 (20) [25]. These Incs, along with those described above, comprise a total of nine Incs preferentially localized to pY419-Src-family kinase enriched microdomains. The precise function of these latter Incs in cytoskeletal dynamics related to extrusion formation remains unknown but collectively, the results support the view of a macromolecular complex with multiple chlamydial components contributing to the process. Deciphering how these multiple proteins interact with the host to control cytoskeletal dynamics will be challenging but informative.

Interestingly, of the nine *C*. *trachomatis* Incs identified in microdomains [24, 25], only four (IncB, IncC, CT288, and CT850) are considered core Incs and are present in all chlamydial species [13]. However, MrcA is not among the conserved Incs [13]. The extrusion mechanism has been reported to be active in multiple chlamydial species [6]. It would be somewhat surprising if entirely different mechanisms were responsible, however, unrecognized chlamydial proteins may supplant the role of those *C*. *trachomatis* Incs not shared among species.

In cell culture models, escape by lysis and by extrusion is approximately equal [6, 20]. The relative frequency of the different escape mechanisms in vivo is unknown. It is suspected that the function of extrusion is for evasion of localized inflammatory responses at the site of infection [6, 20]. Encapsulation of viable EBs within a membrane bound compartment may protect the chlamydia as they disseminate to more distal sites for reinfection. It has recently been shown that extrusions are readily phagocytosed by macrophages where they show enhanced survival and may contribute to dissemination [44]. In vivo evidence of extrusions have also been detected using a cervicovaginal infection model suggesting relevance to active infections [45]. Recognition of the signaling pathways influencing the different exit strategies should provide insights into the regulation of chlamydial dissemination during disease.

## Materials and methods

### Bacterial and cell culture

*Chlamydia trachomatis* serovars L2 (LGV 434/Bu) were propagated in Hela 229 cells (American Type Culture Collections; ATCC CCL-2.1) and EBs were density gradient purified as described [46]. Hela and Vero (ATCC CCL-81) cells were grown in RPMI 1640 medium with 10% fetal bovine serum (FBS) (HyClone Laboratories, Logan, UT) at 37^°^C and 5% CO_2_.

### Yeast-two hybrid screening

Yeast-two hybrid screening was performed with the Matchmaker Gold Yeast-Two Hybrid System (Clontech) according to the manufacturer’s directions with the modifications previously described [20, 41]. Briefly, The C-terminal 297 nucleotides of *mrcA* was PCR amplified with PCR Supermix High Fidelity polymerase (ThermoFisher) using C. trachomatis L2 genomic DNA. The forward primer containing the NcoI site (5’-AAACCATGGCCTCTATCAAACATCGCTTT-3’) and reverse primer containing the BamH1 site (5’-AAAGGATCCGTCAGTAATAATAAACAGA-3’) was used. Amplicons were digested with NcoI and BamHI and ligated into the corresponding restriction sites of pGBKT7 (Clontech) generating pGBKT7-MrcA. Constructs were verified by sequencing. pGBKT7-MrcA was transformed into AH109 and mated with Y187 containing pre-transformed Normalized Mate and Plate HeLa S3 Library (Clontech). Interacting bait and prey partners were identified by selecting diploids on low stringency synthetic dropout (SD) plates lacking leucine (Leu), tryptophan (Trp) and histidine (His). Resulting diploids able to grow on low stringency were rescreened in high stringency conditions on SD plates lacking His, Leu, Trp and adenine supplemented with X-α galactosidase. From yeast diploids that grew in high stringency conditions and turned blue, prey plasmids were extracted using the PrepEase Yeast Plasmid Isolation Kit (Affymetrix). Plasmids were transformed into *E*. *coli* XL1-blue (Agilent Technologies), re-extracted using Qiagen mini plasmid kit and sequenced. Interactions were confirmed with Bait Dependency tests using targeted screens between AH109 carrying pGBKT7-MrcA and Y187 carrying prey plasmids identified in the library screens. Resulting diploids were plated on high stringy SD media and monitored for growth.

### Targetron construction

All restriction enzymes and ligases, phosphatases, and DNA polymerases were purchased from New England Biolabs (NEB, Beverly, MA) unless otherwise specified. Oligonucleotides and primers used in this study were purchased from Integrated DNA Technologies (Skokie, IL) unless otherwise specified. Cloning was performed in *E*. *coli* DH5 alpha MAX Efficiency competent cells (Life Technologies, Carlsbad, CA). The Targetron pACDK4-C plasmid was purchased from Sigma Aldrich (Atlanta, GA) and modified for intron integration in chlamydia as described [29].

The intron was retargeted for *C*. *trachomatis* LGV434/Bu MrcA using the TargeTron computer algorithm (TargeTronics). Insertion sites with the highest score and proximity to the 5’ ATG start codon were selected. Using the primers listed in S1 Table, the intron was retargeted and amplified using a Qiagen core PCR kit (Qiagen). The PCR product was cloned into the BsrGI/HindIII site of pACT, and the ligated plasmid was transformed into methylation-deficient Escherichia coli K-12 ER2925 (New England Biolabs). The integrity of all constructs was verified by sequencing.

### Plasmid construction

*C*. *trachomatis mrcA* was PCR amplified from L2/434/Bu genomic DNA using the primers listed in S1 Table and were cloned into the NotI/SalI site of pBOMB4-Tet-mcherry [28]. Each *orf* was expressed as a C-terminal fusion to a Flag-tag. The integrity of the construct was verified by DNA sequencing.

### Chlamydia transformation

*C*. *trachomatis* serovars L2 was transformed with expression construct as previously described [25, 28]. Briefly plasmid DNA, prepared from methylation deficient *E*. *coli* K12 ER2925 (New England Biolabs) was transformed into *C*. *trachomatis* L2 (LGV434/Bu) density gradient-purified EBs using CaCl_2_ buffer (10mM Tris pH7.5; 50mM CaCl_2_). Following 4 passages under selection with 0.1 U/ml of penicillin G, transformants were plaque cloned in Vero cells and individual plaques were picked and expanded. Disruption of *mrcA* was verified using PCR and sequencing of genomic DNA isolated from plaque-purified bacteria. Expression of the Flag-tagged fusion protein was verified using immunofluorescence microscopy.

### Complementation of the *C*. *trachomatis mrcA::bla* mutant

To complement the *mrcA::bla* mutant, the tetracycline-inducible promoter and Flag-tagged *mrcA* were PCR amplified form pBOMB4-MrcA-Flag using the primers listed in S1 Table. The PCR products were cloned into the SacII/SalI site of pBOMB3 [28, 29] and transformed into *E*. *coli* K-12 ER2925. *MrcA::bla* EBs were transformed with pBOMB3-tet-MrcA-Flag as previously described [29]. Briefly plasmid DNA, prepared from methylation deficient *E*. *coli* K12 ER2925 was transformed into purified *mrcA::bla* EBs using CaCL2 buffer (10mM Tris pH7.5; 50mM CaCl_2_). Following 4 passages under selection with 0.2 μg/ml chloramphenicol, transformants were plaque cloned in Vero cells under selection with 0.8 μg/ml chloramphenicol, and individual plaques were picked and expanded. Expression of the Flag-tagged fusion protein was verified using immunofluorescence microscopy under aTc induction.

### Growth curve analysis

Hela cells were infected on ice with a multiplicity of infection (MOI) of 1. After 30 min on ice, cultures were shifted to 37^°^C to stimulate bacterial uptake. At 0 h, 4 h, 12h, 24h, 36 h, 48 h post-infection, cells were lysed in water and supernatants were applied to fresh Hela cells monolayers to enumerate inclusion-forming units (IFUs).

### Protein preparation and western blotting

Hela cells were infected with *C*. *trachomatis* at a MOI of 1. At appropriate times post-infection, cells were rinsed in phosphate-buffered saline (PBS, pH 7.4) and lysed in Laemmli buffer [47] with 5% ß-mercaptoethanol. Protein extracts and cell lysates were separated by SDS-PAGE and transferred to nitrocellulose. Membranes were blocked in 5% nonfat dry milk in TBS-T (50mM Tris-HCl, pH7.4; 150mM NaCl; 0.1% Tween 20) and incubated with protein specific primary antibody in blocking buffer overnight at 4°C. Blots were rinsed 3 times for 5 min in TBS-T and incubated with HRP-conjugated secondary antibody. Blots were developed with Amersham ECL prime western blotting detection reagent (GE Healthcare Life Science, Pittsburgh, PA) and exposed to CL-X posure film (Thermo Scientific, Rockville, MD).

### Coimmunoprecipitation

Hela cells were mock infected or infected with transformed *C*. *trachomatis* L2. At 48 hrs postinfection, cells were washed with cold PBS, scraped into 1ml of RIPA buffer plus Complete mini protease inhibitor cocktail (Roche) and incubated on ice for 20 min. Cells debris was removed by centrifugation (13,000 rpm, 5 min, 4°C) and the lysate was pre-clarified by rotation for 1 h at 4°C with 5μl protein-A agarose beads (Cell Signaling, Beverly, MA). Protein-A beads were incubated with rabbit antibody against ITPR3 for 4 hrs at room temperature. The beads were rinsed 3 times with wash buffer (20mM Tris-HCl, pH7.5; 1mM EDTA; 150mM NaCl; 1% NP-40 and protease inhibitors) and mixed with the pre-cleared cell lysate. After gentle agitation overnight at 4°C, the beads were washed 3 times and proteins were eluted into 1x SDS-PAGE sample buffer then boiled at 100°C for 5 min. The eluted proteins were separated by SDS-PAGE on a 12% acrylamide gel for immunoblotting.

#### siRNA knockdown

Hela cells were plated to approximately 50% confluency in 24-well plates, or on glass coverslip for immunostaining. Cells were transfected with Targetplus Smartpool siRNA corresponding to ITPR3 or non-targeting sequence (Dharmacon, Lafayette, CO) according to the manufacturer’s instructions. At 72 hrs post-transfection, cells were infected with *C*. *trachomatis* L2 at an MOI of 1. Cells were fixed with methanol for immunostaining at 24 hrs post infection. The extrusions were enumerated at 48 hrs post infection.

### Immunofluorescence

Hela cells were seeded onto glass coverslips and after 24 hrs were infected at a MOI of 1. At 24 hrs post infection, cells were fixed with methanol and blocked using 1% BSA in TBS-TX (25mM Tris-HCl, pH7.5; 150mM NaCl; 0.1% Triton X-100). Cells were stained with appropriate primary antibodies which were observed by staining with goat anti-rabbit IgG-DyLight 594 and goat anti mouse IgG-DyLight488 secondary antibodies (Jackson Immunoresearch). Images were captured on a Nikon Eclipse 80i fluorescence microscope and analyzed using Nikon Elements software.

### Extruded inclusion enumeration

Extrusion inclusions were enumerated by a method similar to that of Chin et al [20, 48]. Briefly, Hela cells were infected with *C*. *trachomatis* at an MOI ~1. At the indicated times, the supernatants were removed and gently pelleted by centrifugation at 1200 rpm, 20 minutes. The pellet was resuspended in 100 μl of media, mixed with trypan blue (Life Technologies) and stained with Nucblue Hoeschst live cell stain (Life Technologies). Extrusions free of host cell nuclei were enumerated using a Hausser Bright-line Phase hemocytometer and reported as extrusions/ml.

## Supporting information

S1 Fig Growth curve of *C*. *trachomatis* L2 transformed with pBOMB4-MrcA-Flag and localization of MrcA-Flag in inclusion microdomains. (A) Hela cells infected with wild-type L2 or L2 transformed with pBomb4-MrcA-Flag at MOI of 1 were incubated for 0 h, 4 h, 12 h, 24 h, 36 h, or 48h. Cells were lysed in water and replated on fresh monolayers to enumerate the progeny IFUs. (B) Indirect immunofluorescence, Hela cells were infected with *C*. *trachomatis* L2 transformed with pBOMB4-MrcA-Flag for 8 h, at which time 50ng/ml of anhydro-tetracycline hydrochloride were added to induce the expression of MrcA-Flag. At 24 hpi, cells were fixed and stained with anti-Flag (red) and anti-MOMP (green). Bar = 10 μm.
(TIF)

S2 Fig Inclusion membrane microdomains coincide with ER-membrane contact sites. Hela cells monolayers were infected with *C*. *trachomatis* L2 at an MOI of 1 for 24hpi. Cells were fixed and labeled with anti-pY416-Src [24] and anti-CERT [18]. Other images show STIM1 (red) [30] co-localization with MrcA (green) using a rabbit polyclonal anti-MrcA antibody [24] or pY464-MLCK (green) [20]. Bar = 10 μm.
(TIF)

S3 Fig Effects of caffeine on extrusion formation. Wild-type L2 infected cells were treated with 200, 400 and 800μg/ml of caffeine. (A) At 48 hrs post infection, extruded inclusions were enumerated (n = 3, error bars represent SEM). Differences were significant at levels of p<0.01; ** or p<0.005; *** by an unpaired Students T-test. (B) Cells were lysed in water and replated on fresh Hela cells monolayer to enumerate IFUs. (C) Indirect immunofluorescence, L2 infected cells were treated with caffeine [28] (400μg/ml). At 18 hrs post infection, infected cells were fixed and stained with anti-pT853-MYPT1, anti-pS19-MLC2 (red) and anti-MOMP (green) primary antibodies. Bar = 10 μm. (D) Western blot analysis of infected cells treated by caffeine (400μg/ml).
(TIF)

S1 Table Primers used in this study. (DOCX)
