# Supplementary figures and images for: Chlamydia trachomatis inclusion membrane protein MrcA interacts with the inositol 1,4,5-trisphosphate receptor type 3 (ITPR3) to regulate extrusion formation
Source: PLoS Pathog. 2018 Mar 15;14(3):e1006911. doi: 10.1371/journal.ppat.1006911 (PMC5854415; doi:10.1371/journal.ppat.1006911)

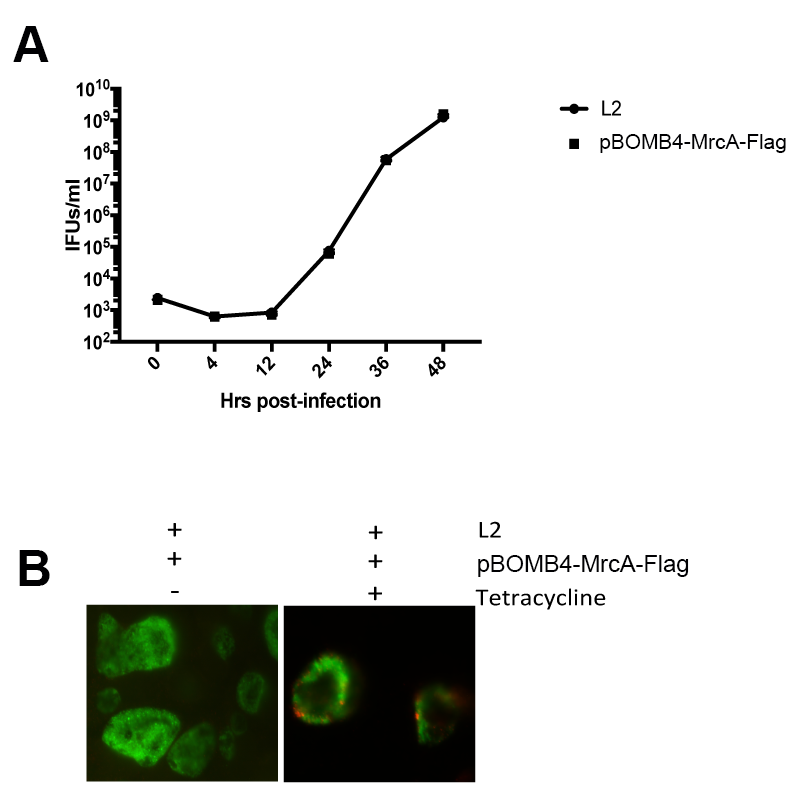

Supplement: S1 Fig — (A) Hela cells infected with wild-type L2 or L2 transformed with pBomb4-MrcA-Flag at MOI of 1 were incubated for 0 h, 4 h, 12 h, 24 h, 36 h, or 48h. Cells were lysed in water and replated on fresh monolayers to enumerate the progeny IFUs. (B) Indirect immunofluorescence, Hela cells were infected with C. trachomatis L2 transformed with pBOMB4-MrcA-Flag for 8 h, at which time 50ng/ml of anhydro-tetracycline hydrochloride were added to induce the expression of MrcA-Flag. At 24 hpi, cells were fixed and stained with anti-Flag (red) and anti-MOMP (green). Bar = 10 μm. (TIF) [file ppat.1006911.s001.tif]

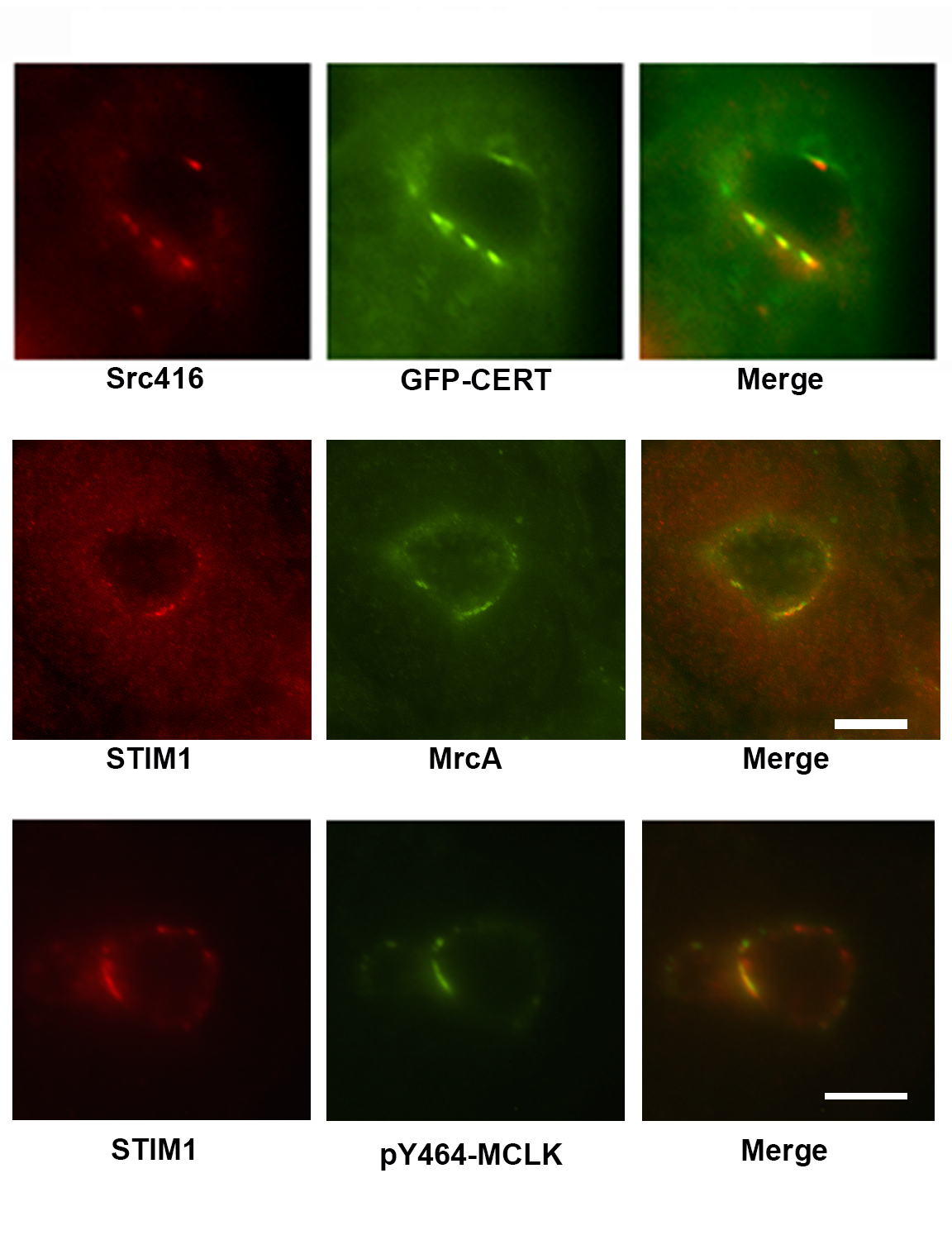

Supplement: S2 Fig — Hela cells monolayers were infected with C. trachomatis L2 at an MOI of 1 for 24hpi. Cells were fixed and labeled with anti-pY416-Src [24] and anti-CERT [18]. Other images show STIM1 (red) [30] co-localization with MrcA (green) using a rabbit polyclonal anti-MrcA antibody [24] or pY464-MLCK (green) [20]. Bar = 10 μm. (TIF) [file ppat.1006911.s002.tif]

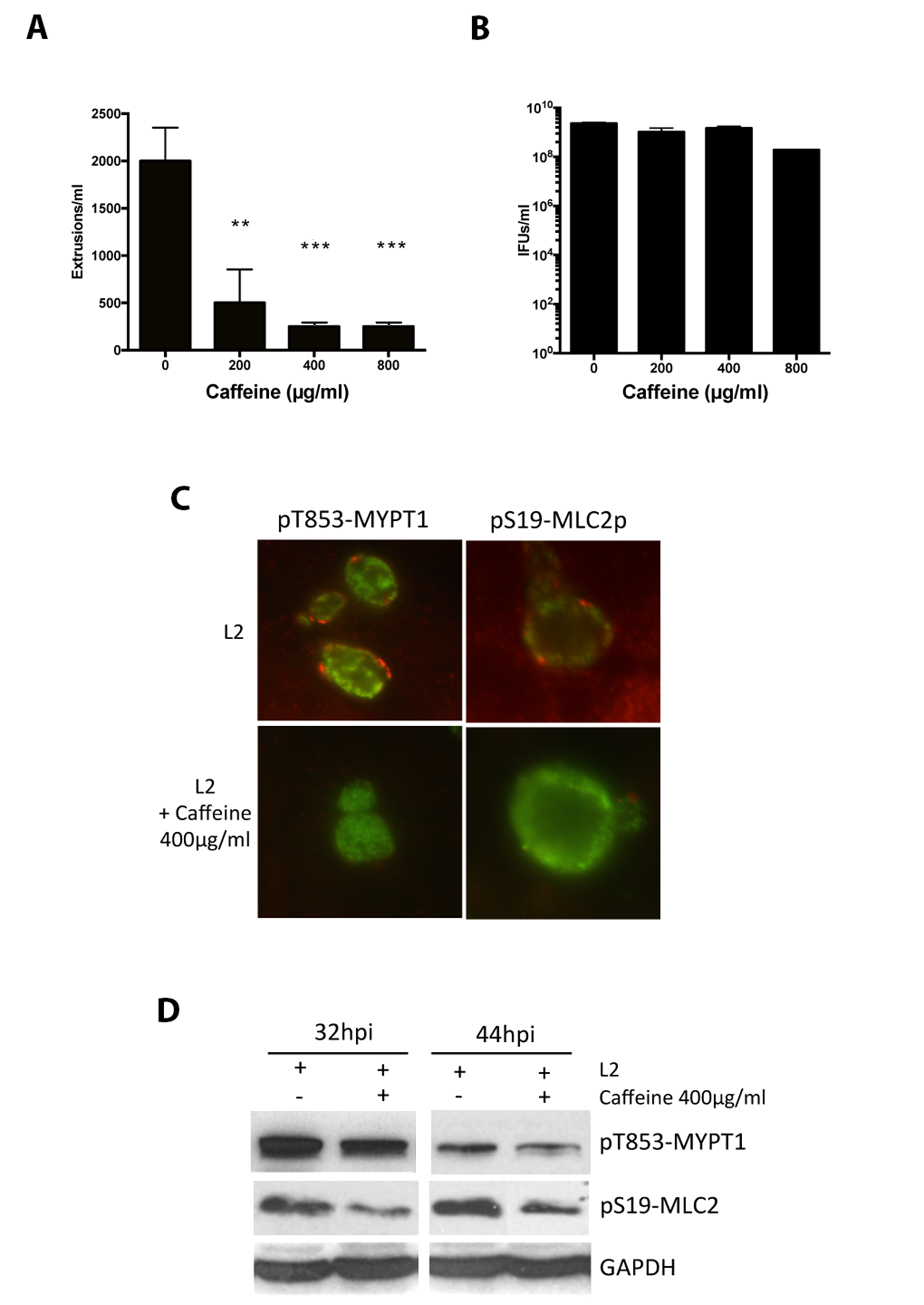

Supplement: S3 Fig — Wild-type L2 infected cells were treated with 200, 400 and 800μg/ml of caffeine. (A) At 48 hrs post infection, extruded inclusions were enumerated (n = 3, error bars represent SEM). Differences were significant at levels of p<0.01; ** or p<0.005; *** by an unpaired Students T-test. (B) Cells were lysed in water and replated on fresh Hela cells monolayer to enumerate IFUs. (C) Indirect immunofluorescence, L2 infected cells were treated with caffeine [28] (400μg/ml). At 18 hrs post infection, infected cells were fixed and stained with anti-pT853-MYPT1, anti-pS19-MLC2 (red) and anti-MOMP (green) primary antibodies. Bar = 10 μm. (D) Western blot analysis of infected cells treated by caffeine (400μg/ml). (TIF) [file ppat.1006911.s003.tif]

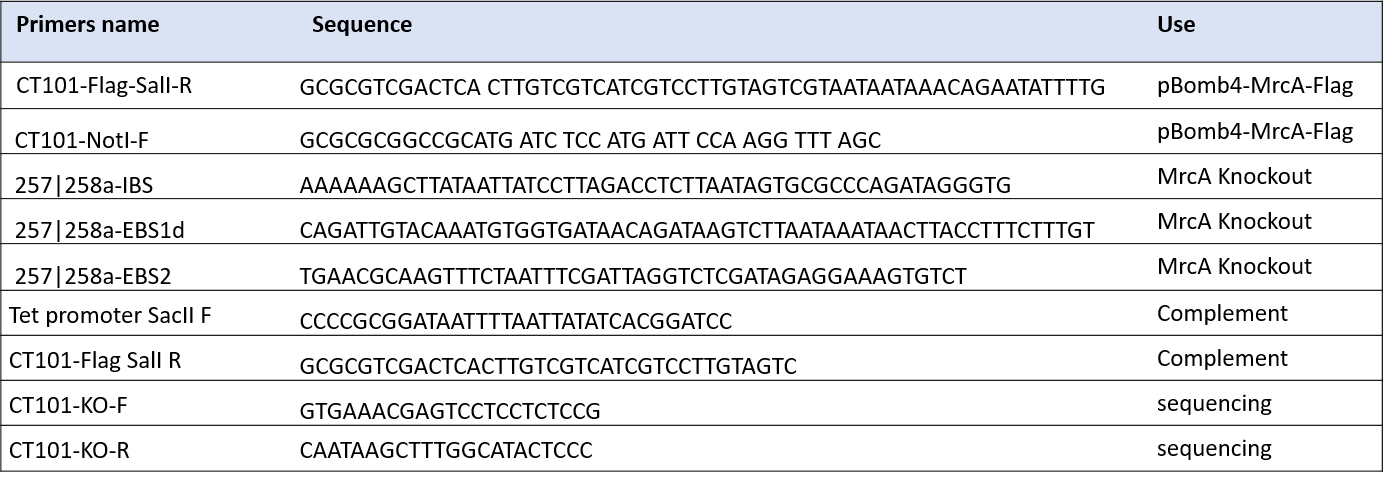


Table S1. Primers used in this study

Supplement: S1 Table — (DOCX) [file ppat.1006911.s004.docx]
